# Supplementary material for: Effect of Microbial Inoculation on Carbon Preservation during Goat Manure Aerobic Composting
Source: Molecules. 2021 Jul 23;26(15):4441. doi: 10.3390/molecules26154441 (PMC8348721; doi:10.3390/molecules26154441)
Supplement: Supplementary file 1 [file molecules-26-04441-s001.zip › molecules-1282178-supplementary.pdf]

Supporting Materials

Effect of Microbial Inoculation on Carbon Preservation During Goat Manure Aerobic Composting

Jiawei Lu [1], Jingang Wang [2], Qin Gao [1], Dongxu Li [1], Zili Chen [1], Zongyou Wei [3], Yanli Zhang [1,2,*] and Feng Wang [1,2,*]

[1] Institute of Goats and Sheep Science, Nanjing Agricultural University, NO. 1 Weigang, Nanjing, 210095, China
[2] Research Centre of Haimen oats, Nanjing Agricultural University, Haimen, Jiangsu 216121, China
[3] Taicang Agricultural and Rural science & Technology Service Center, Taicang, Jiangsu 215400, China

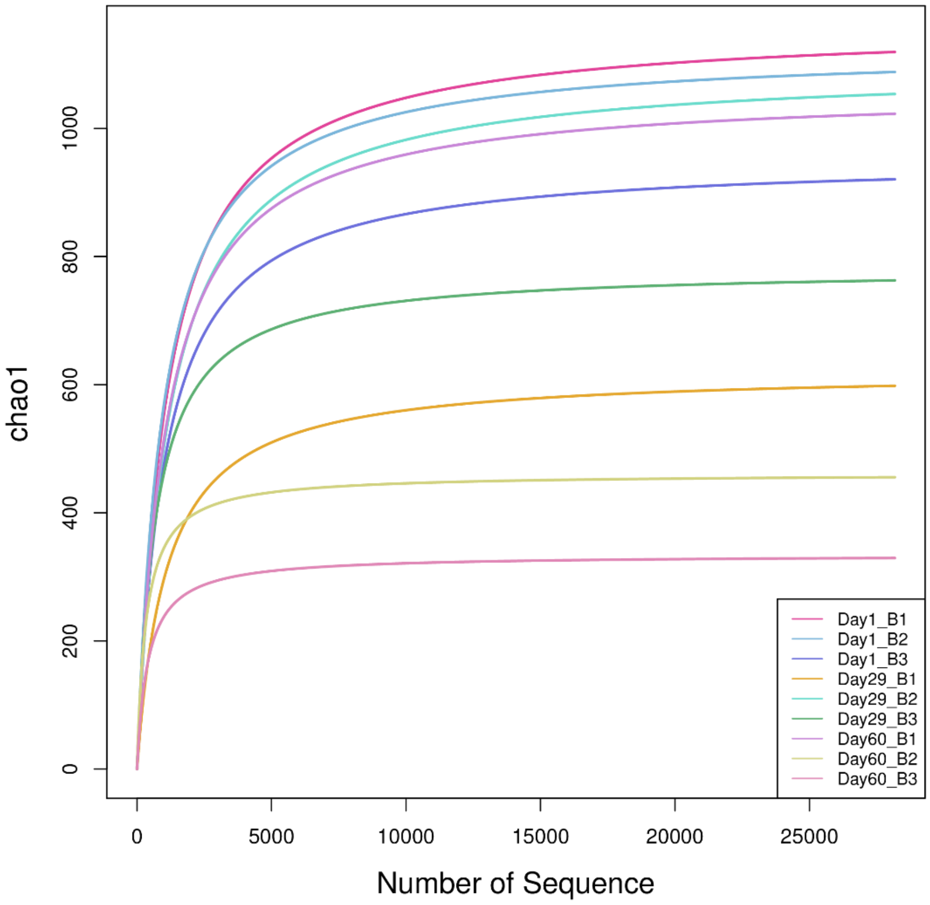

Figure S1. Chao 1 index.

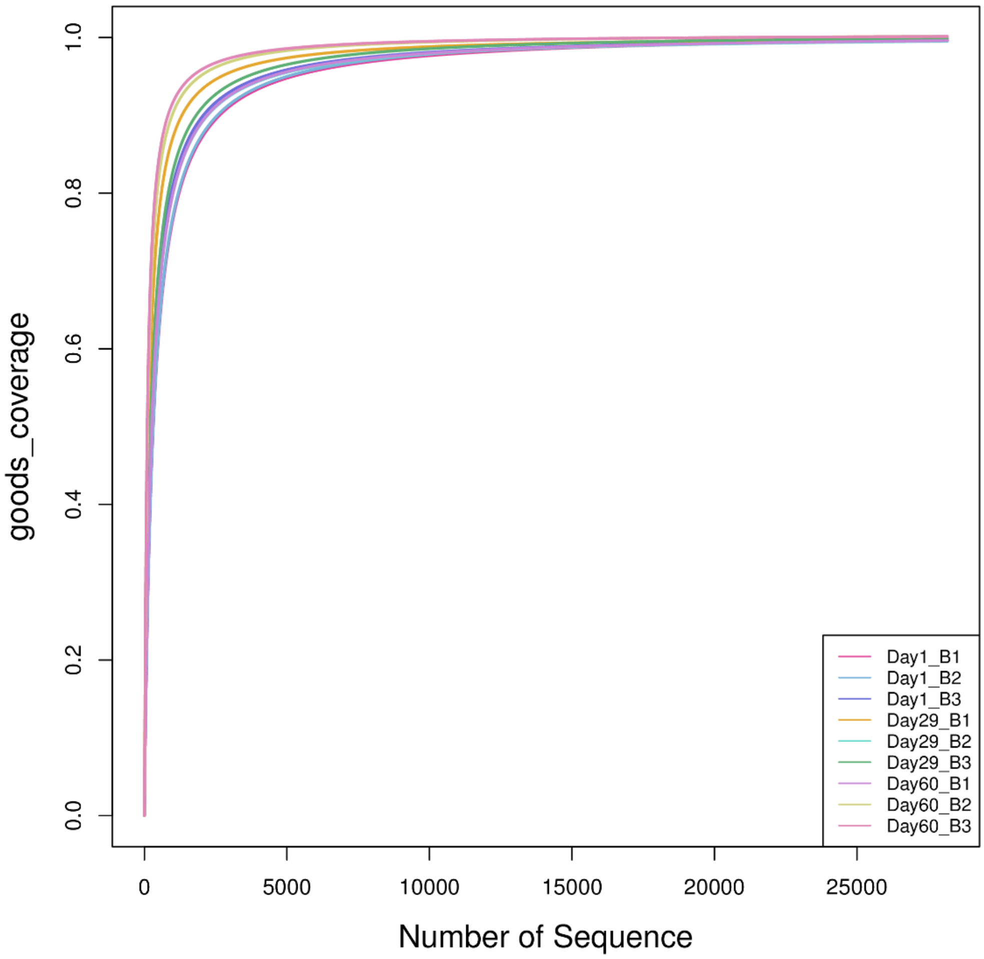

Figure S2. Goods_coverage index.

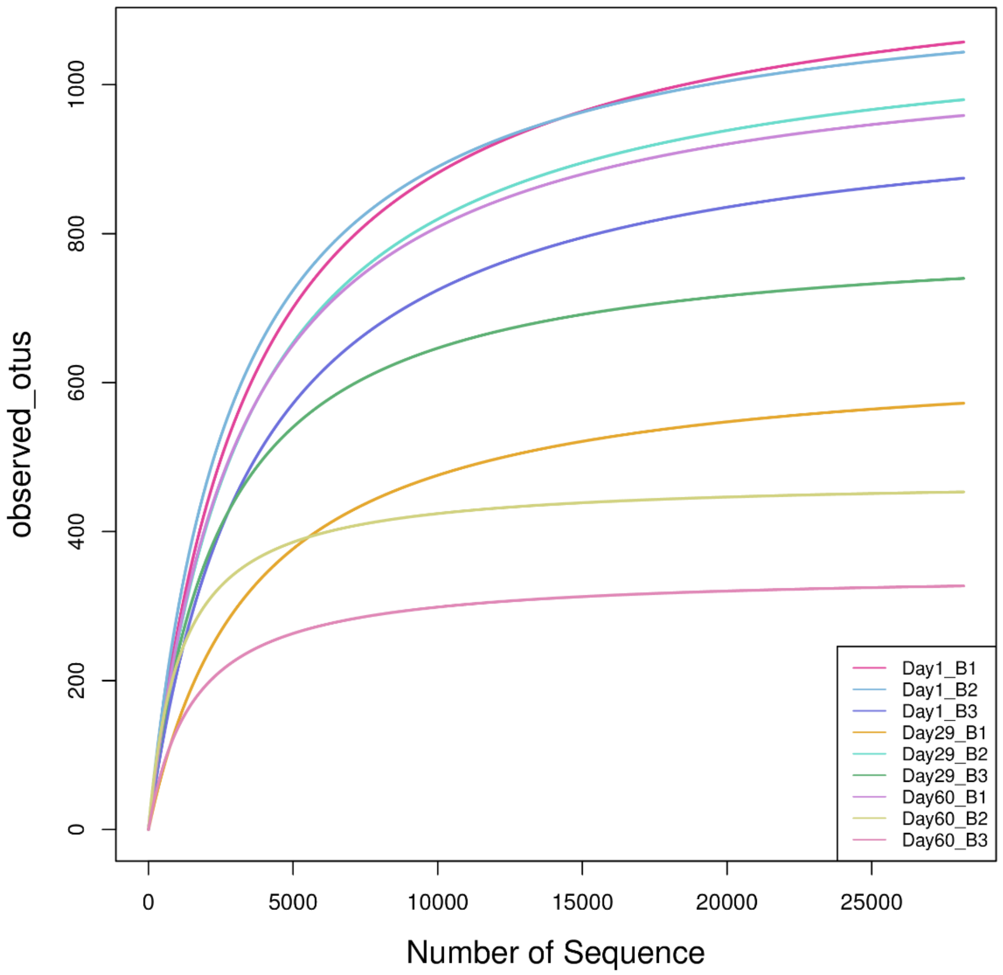

Figure S3. Observed_otus index.

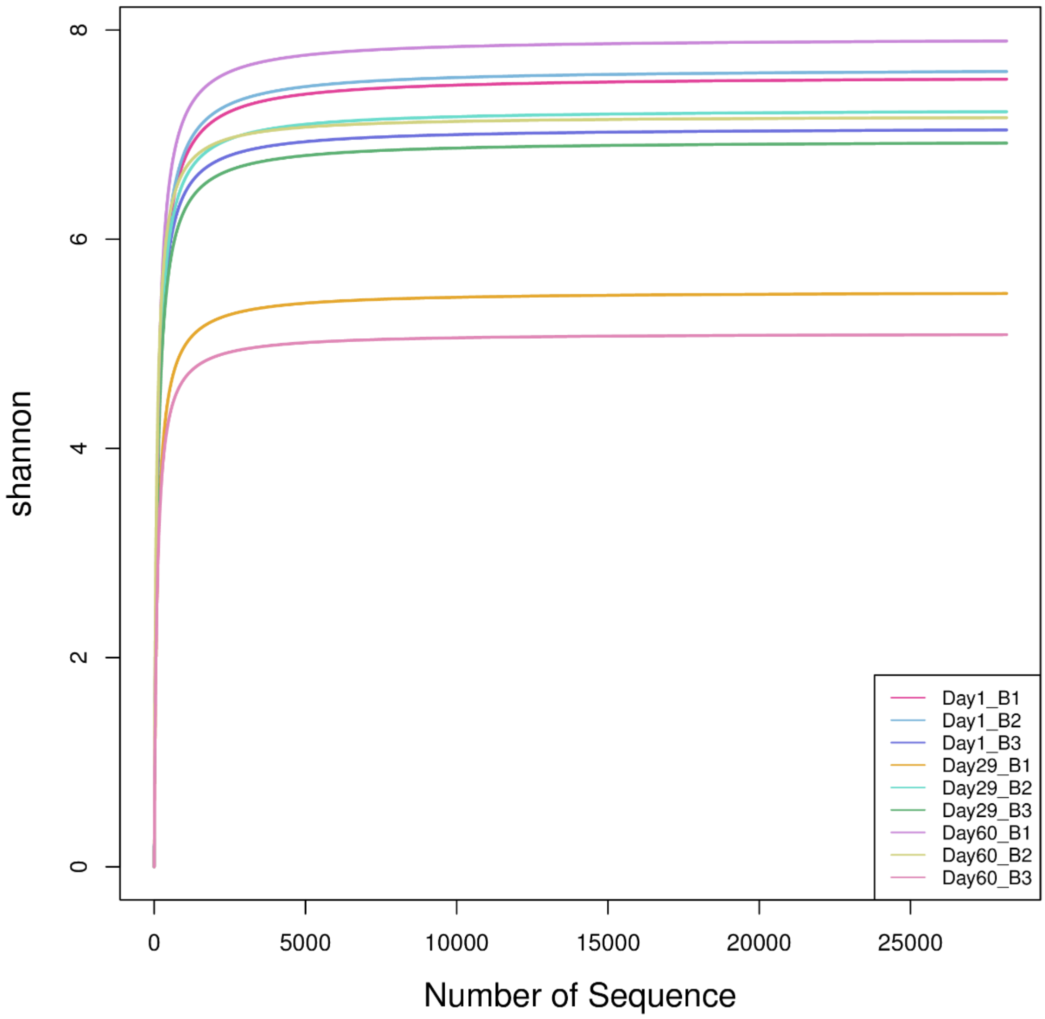

Figure S4. Shannon index.

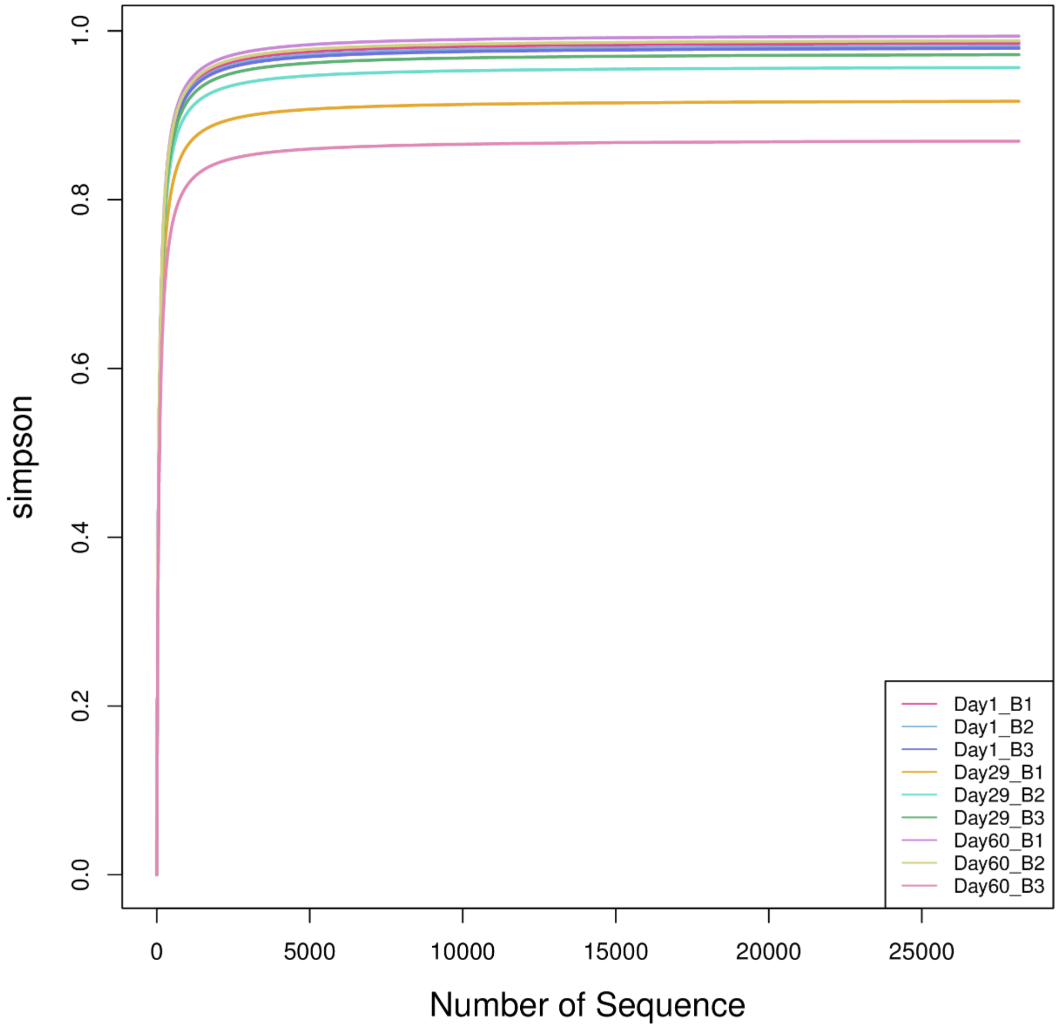

Figure S5. simpson index.

Table S1. Basic data.

Sample	Raw_tags	Raw_Bases	Valid_Tags	Valid _Bases	Valid%	Q20%	Q30%	GC%
Day1_B1	83968	41.98M	72433	30.35M	86.26	97.79	93.29	53.08
Day1_B2	81010	40.51M	63621	26.74M	78.53	97.23	91.93	52.88
Day1_B3	63750	31.88M	58125	24.40M	91.18	97.29	92.23	51.52
Day29_B1	60724	30.36M	55325	23.41M	91.11	96.91	91.35	51.98
Day29_B2	84765	42.38M	66855	27.62M	78.87	97.18	91.67	54.14
Day29_B3	59811	29.91M	55728	23.32M	93.17	97.49	92.67	50.91
Day60_B1	84695	42.35M	68419	28.26M	80.78	97.48	92.68	53.37
Day60_B2	83311	41.66M	68685	28.54M	82.44	94.52	86.14	52.74
Day60_B3	70579	35.29M	55222	22.87M	78.24	94.49	85.39	56.23
